# Genetic Diagnosis of Rubinstein–Taybi Syndrome With Multiplex Ligation-Dependent Probe Amplification (MLPA) and Whole-Exome Sequencing (WES): Case Series With a Novel *CREBBP* Variant

**DOI:** 10.3389/fgene.2022.848879

**Published:** 2022-04-08

**Authors:** Yu-Rong Lee, Yu-Chen Lin, Yi-Han Chang, Hsin-Yu Huang, Yi-Kai Hong, Wilson Jr F. Aala, Wei-Ting Tu, Meng-Che Tsai, Yen-Yin Chou, Chao-Kai Hsu

**Affiliations:** ^1^ Department of Dermatology, National Cheng Kung University Hospital, College of Medicine, National Cheng Kung University, Tainan, Taiwan; ^2^ School of Medicine, College of Medicine, National Cheng Kung University, Tainan, Taiwan; ^3^ International Center of Wound Repair and Regeneration, College of Medicine, National Cheng Kung University, Tainan, Taiwan; ^4^ Education Center, National Cheng Kung University Hospital, College of Medicine, National Cheng Kung University, Tainan, Taiwan; ^5^ Institute of Clinical Medicine, College of Medicine, National Cheng Kung University, Tainan, Taiwan; ^6^ Department of Pediatrics, National Cheng Kung University Hospital, College of Medicine, National Cheng Kung University, Tainan, Taiwan

**Keywords:** rubinstein-taybi syndrome, multiplex ligation-dependent probe amplification, whole-exome sequencing, next-generation sequencing, genetic diagnosis, novel variant, CREBBP (Crebb binding protein)

## Abstract

Rubinstein–Taybi Syndrome (RSTS) is a rare congenital disease with distinctive facial features, broadening of the thumbs and halluces, and developmental delay. RSTS is caused by *de novo* genetic alterations in *CREBBP* and the homologous *EP300* genes. In this study, we established a genetic diagnostic protocol by integrating multiplex ligation-dependent probe amplification (MLPA) and whole-exome sequencing (WES). Five patients clinically diagnosed with RSTS were enrolled for genetic testing. Germline DNA was extracted from the peripheral blood of the patients and their families. One patient (case 1) was identified as harboring a large heterozygous deletion in the 16p13.3 region, spanning the *CREBBP* gene. Three patients (Cases 2–4) harbored different *CREBBP* variants (c.2608C>T:p.Gln870Ter,c.4404_4405del:p.Thr1468fs,c.3649C>T:p.Gln1217Ter). No causative variants were identified for the fifth RSTS patient (case 5). Here, we propose a molecular diagnostic protocol that identified causative genetic alterations in 4/5 of the patients, yielding a molecular diagnostic rate of 80%. Given the rarity of RSTS, more research is needed to explore its pathogenesis and mechanism.

## Introduction

Rubinstein–Taybi Syndrome (RSTS, Broad thumb–hallux syndrome, MIM 180849) is a rare congenital malformation syndrome that affects approximately 1 in 100,000–125,000 newborns. RSTS is characterized by the broadening of the thumbs and halluces, developmental delay, moderate to severe intellectual disability, proneness to keloids, and distinctive facial features such as a large beaked nose and a low-hanging columella (Roelfsema and Peters, 2007).

Genetic research on RSTS has mainly focused on mutations in the genes encoding CREB binding protein (*CREBBP*, MIM 600140) and its homolog, E1A-binding protein P300 (*EP300*, MIM 602700). *CREBBP* is located at 16p13.3, while *EP300* is located at 22q13.2. Both CBP and p300 are highly homologous transcriptional coactivators sharing conserved protein–protein interaction domains including the enzymatic histone acetyltransferase (HAT) domain. Acetylation disrupts the DNA–histone interaction by neutralizing the positively charged lysine residues in histones and enabling increased accessibility for transcription factors to activate gene expression (Kalkhoven, 2004; Oliveira et al., 2006). Among patients with RSTS, roughly 50–60% harbor pathogenic variants in *CREBBP* (RSTS type I), while only 3–8% of the patients harbor mutations in *EP300* (RSTS type II) (López et al., 2018). The vast majority (about 99%) of RSTS cases occur sporadically from *de novo* heterozygous *CREBBP* mutations, although vertical transmission has also been documented in a handful of cases (Bartsch et al., 2010). The spectrum of reported genetic alterations regarding RSTS includes point mutations (i.e., frameshift, nonsense, missense, and splice-site mutations), intragenic or large deletions, translocations and inversions (Negri et al., 2019).

Whole-exome sequencing (WES), which utilizes the “shotgun-based” approach of next-generation sequencing, is known for its efficiency and effectiveness in detecting single-nucleotide polymorphisms (SNPs) and small insertions/deletions (indels). Structural variants and large deletions, on the other hand, remain challenging for WES due to its reliance on the short-read approach (Chong et al., 2015; Chiu et al., 2021). Multiplex ligation-dependent probe amplification (MLPA), on the other hand, although unable to recognize SNPs and indels, shows strengths in detecting genomic variations, such as copy number variations (CNVs) and large-spanning deletions by comparing the differences in PCR-amplified fluorescently labeled primers binding to the probes. Although molecular diagnosis can reveal the genetic alterations in most RSTS cases, a sizeable subset of patients (30%) remain undiagnosed when this method is used (Tajir et al., 2013). In this study, we leveraged the distinct advantages of WES and MLPA by combining the two methods in our genetic diagnostic protocol for RSTS. Here, we report three patients with pathogenic *CREBBP* mutations and one with a large deletion spanning *CREBBP* in a five-patient cohort of clinically diagnosed RSTS individuals.

## Materials and Methods

### Patients

The inclusion criteria included concurrent presentation of common RSTS clinical characteristics (distinctive facial features, broadening of the thumbs and halluces, short stature) unexplained by other systemic syndromes (Figure 2). Five patients (3 males and 2 females) fulfilled the criteria and were enrolled for further molecular genetic diagnosis. Detailed clinical data of the cases were documented. 3/5 (60%) of the cases also had microcephaly, another common presentation of RSTS. Additional clinical findings relevant to RSTS, including cyanotic heart disease, pulmonary hypertension, intellectual disability, and other neurological impairments, were found in some cases. The clinical manifestations of each case enrolled in this study are summarized in Table 1. Ethics approval was granted by the Ethics Committee of National Cheng Kung University Hospital (A-BR-104-052). Informed consent was obtained from the patients’ parents. Peripheral blood specimens were collected from the patients. DNA was extracted using the Qiagen FlexiGene DNA Kit (Qiagen, Hilden, Germany) according to the manufacturer’s instructions.

**TABLE 1:** Summary of causative genetic alterations found in this study.

Case	Gender	Age at diagnosis (months)	Phenotype Description at Diagnosis	Molecular Genetic Diagnosis	
				MPLA	WES
Case 1	Female	1	Short stature^a^, microcephaly^b^, cyanotic congenital heart disease, pulmonary hypertension, prominent nose, broad thumbs and/or halluces, keloids	16p13.3 (3,728,096-3,962,938)del	Low read coverage across the mutation span
Case 2	Male	36	Prominent nose, mild intellectual disability, mild mental retardation, broad thumbs and/or halluces, keloids	Normal^c^	*CREBBP:exon14:c.2608C>T:p.Gln870Ter (CADD:21.4, DANN:0.9986)
Case 3	Female	27	Short stature^a^, microcephaly^b^, broad thumbs and/or halluces	Normal^c^	*CREBBP*:exon27:c.4404_4405del:p.Thr1468fs (CADD:NA, DANN:NA)
Case 4	Male	8	Short stature^a^, ventricular septal defect, patent ductus arteriosus, hearing loss, bilateral hearing impairment, cryptorchidism, broad thumbs and/or halluces	Normal^c^	*CREBBP*:exon19:c.3649C>T:p.Gln1217Ter (CADD:44, DANN:0.9987)
Case 5	Male	32	Short stature^a^, microcephaly^b^, congenital glaucoma, broad thumbs and/or halluces	Normal^c^	Normal^d^

a ≤3rd percentile for height.

b ≤3rd percentile for occipital frontal circumference.

c No large intragenic deletions detected in CREBBP or EP300.

d No pathogenic SNP or indels detected.

*novel variant.

### Multiplex Ligation-Dependent Probe Amplification

MLPA was performed on DNA from each patients via the Affymetrix 750K array platform (Thermo Fisher Scientific, Waltham, United States) using a human microdeletion syndrome probe set (SALSA P096; MRC-Holland, Amsterdam, Netherlands). On the whole, microdeletions in the 4p, 5p15, 8p, 8q24, 11p13-14, 16p13.3, and 21q22.2 regions were detected with the probe set and were thus analyzed. MLPA reveals the relative quantification of the number of copies of targeted DNA sequences. Data were analyzed using GeneMarker software (SoftGenetics, State College, PA, United States). For the purpose of this study, heterozygous or homozygous deletions in the 16p13.3 region, which spans the *CREBBP* gene, were considered abnormal positives.

In one patient showing an abnormal large deletion spanning the *CREBBP* gene (case 1), MLPA was also done on the germline DNA from the parents of the proband to examine whether the mutation occurs *de novo*. As for the patients showing negative results for *CREBBP* large deletions, WES was performed on the DNA specimen to detect pathogenic SNPs and indels in the genes *CREBBP* and *EP300*.

### Whole-Exome Sequencing and Sanger Sequencing

Germline DNA extracted from the probands was used for paired-end library preparation using the SureSelect All Exon 50 Mb Version 4.0 kit (Agilent, Santa Clara, CA, United States) according to the manufacturer’s recommendations. Sequencing was carried out by massively parallel sequencing with 100-bp paired-end reads using the HiSeq-2000 platform (Illumina, CA, United States). The Novoalign software package (Novocraft Technologies Sdn Bhd) was used to align reads generated to the reference human genome. Reads mapping to multiple locations on the reference human genome were excluded from downstream analysis. The BedTools package was used to calculate the depth and breadth of sequence coverage (Quinlan and Hall, 2010). Single-nucleotide substitutions and small indels were detected with the SamTools package (Li et al., 2009). Sequence variants were annotated with the Annovar tool (Wang et al., 2010). To assess the pathogenicity of the candidate variants, an in-house variant-filtering pipeline was used. Nonsense variants or indels resulting in frame shifts in *CREBBP* or *EP300* with minor allele frequencies (MAF) of less than 0.5% in the 1,000 Genomes Project (Siva, 2008) and Exome Aggregation Consortium (ExAC) were included. The damage prediction criteria for filtering the candidate variants included a Combined Annotation Dependent Depletion (CADD) score of above 15, a Deleterious Annotation of Genetic variants using Neural Networks (DANN) score of above 0.95, and a Polymorphism Phenotyping v2 (PolyPhen-2) score of above 0.95. Variants with MAF exceeding 0.5% or with damage prediction scores not fulfilling our criteria were excluded as non-pathogenic.

BAM files of WES were visualized via Integrative Genomics Viewer (IGV) (Robinson et al., 2011). Confirmative polymerase chain reaction (PCR) and Sanger sequencing tests were performed on the DNA from the probands and their parents to validate the filtered variants detected by WES and for segregation analysis. Primers were designed using the Ensembl database (Howe et al., 2021) and Primer3 (Untergasser et al., 2012) online software. The original contributions presented in the study are publicly available. This data can be found here: NCBI, PRJNA806385.

## Results

**FIGURE 1:**
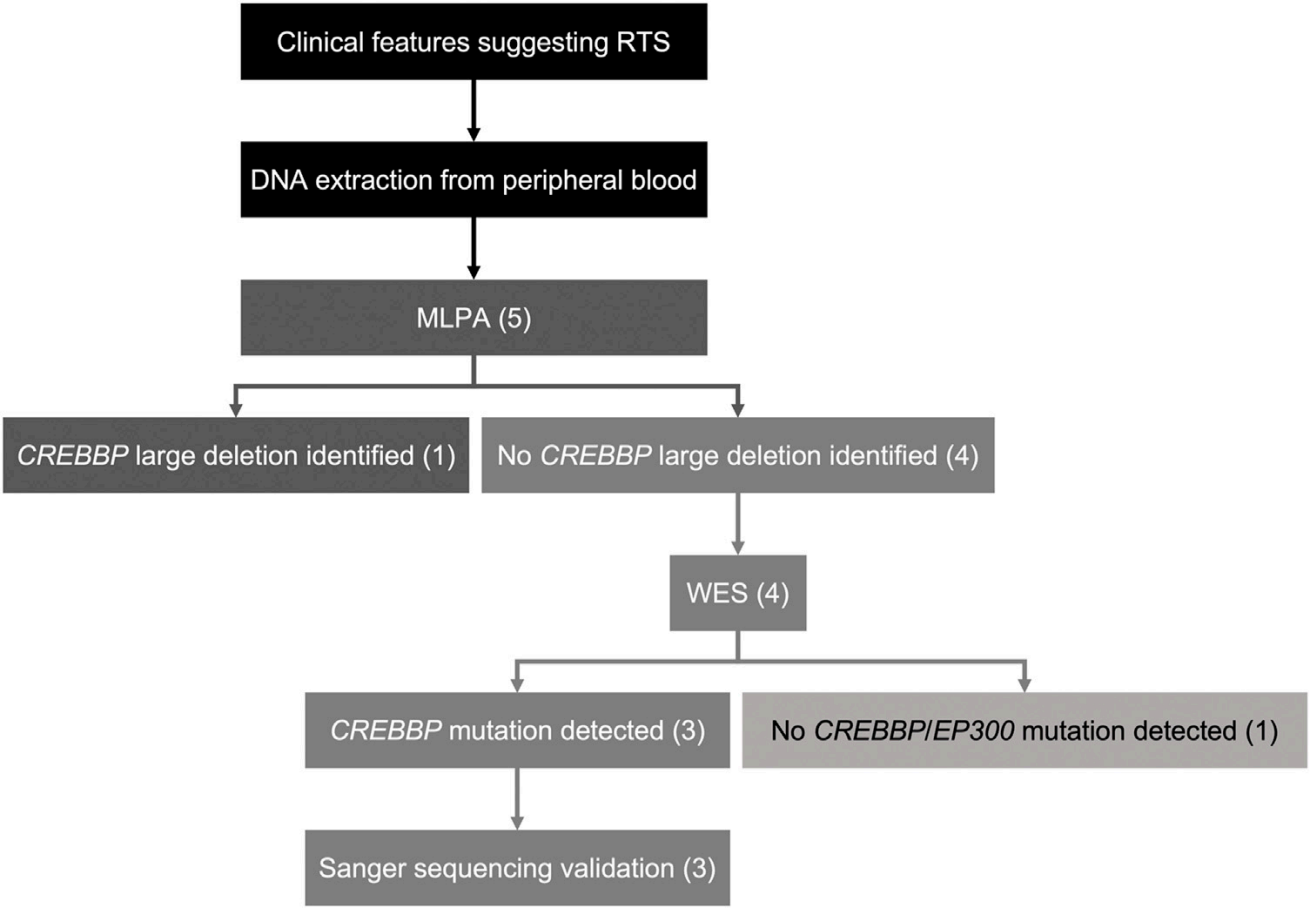
Schematic of the diagnostic workflow in this study. Five patients clinically diagnosed with RSTS were enrolled. MLPA was done on DNA samples of all the RSTS patients. 1/5 patient (20%) was found to harbor a large *de novo* deletion spanning *CREBBP*. WES was performed on DNA samples of the other four patients showing negative MLPA results. 3/5 patients (60%) were found to have novel *CREBBP* mutations with high pathogenicity scores. 1/5 patient (20%) showed negative results for MLPA and WES.

**FIGURE 2:**
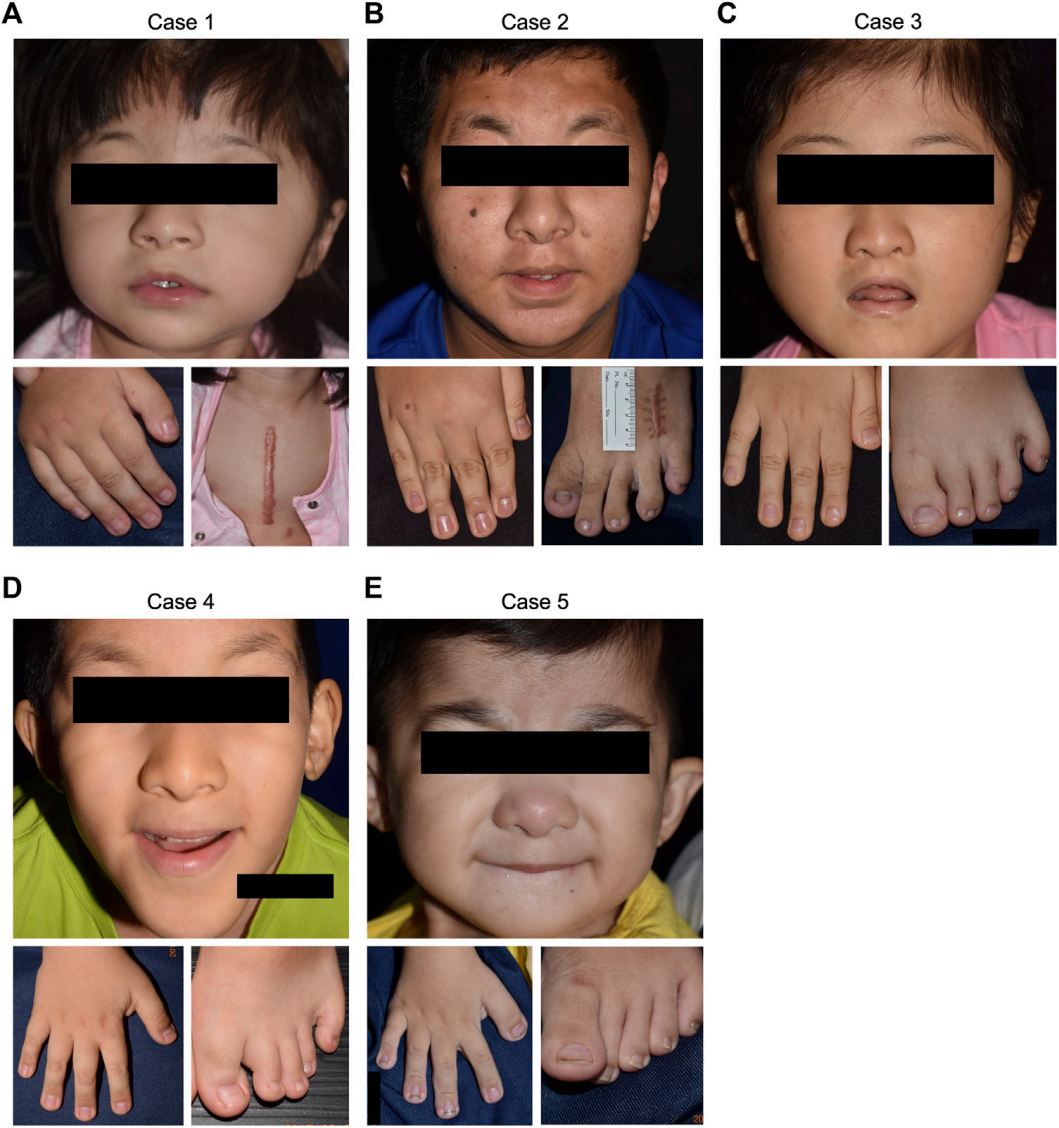
Clinical manifestations of the five RSTS cases. Positive findings of common RSTS characteristics, including distinctive facial features, and broadened thumbs and halluces, were noted in all of the enrolled cases. **(A)** case 1. **(B)** case 2. **(C)** case 3. **(D)** case 4. **(E)** case 5.

Genetic alterations relevant to RSTS identified in this study by both MLPA and WES are summarized in Figure 1. The characteristic clinical manifestations of RSTS for all five cases are summarized in Figure 2. As a whole, among the patients enrolled, MLPA showed a large 16p13.3 DNA deletion spanning the *CREBBP* gene in 1/5 (20%) patient, while causative *CREBBP* variants were detected in 3/5 (60%) patients by WES and were confirmed by Sanger sequencing. Through MLPA and WES, causative variants were identified in 4/5 (80%) of the RSTS patients and are summarized in Table 1.

**FIGURE 3:**
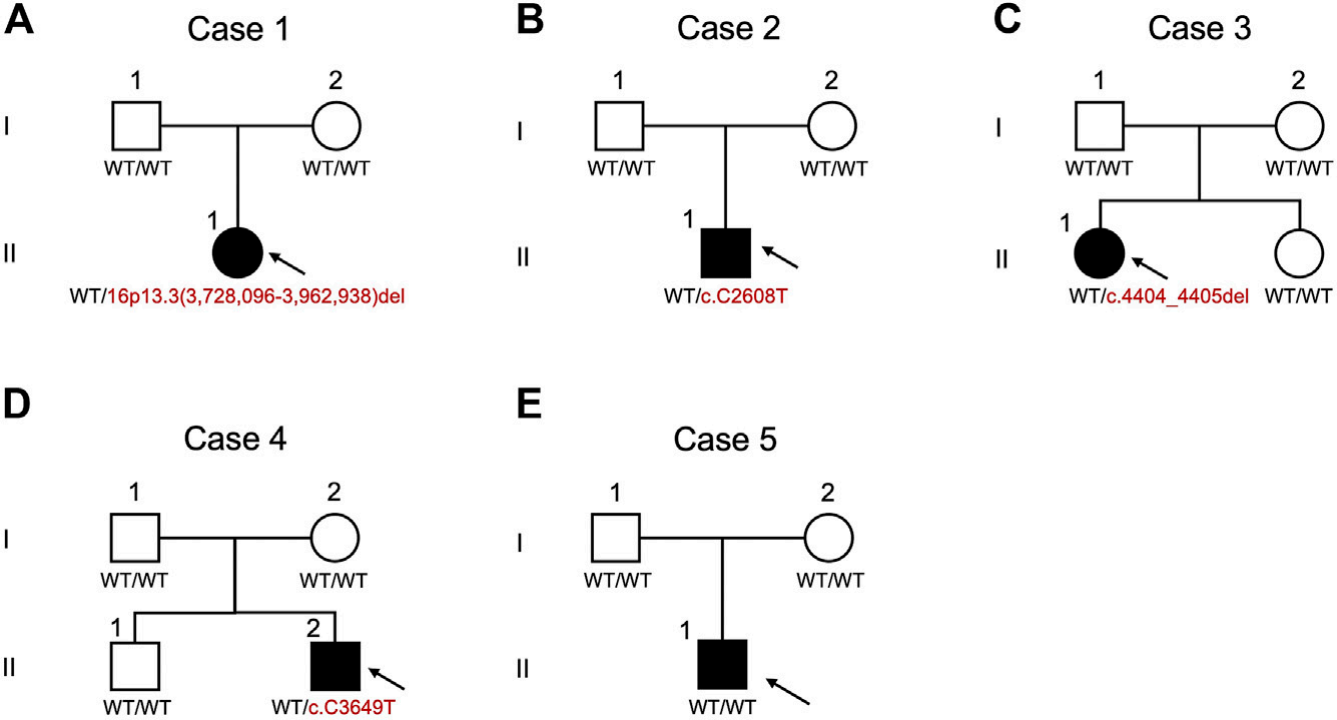
Pedigrees and schematic summary of genetic alteration discoveries in this study. **(A)** case 1. **(B)** case 2. **(C)** case 3. **(D)** case 4. **(E)** case 5. WT, wild type.

**FIGURE 4:**
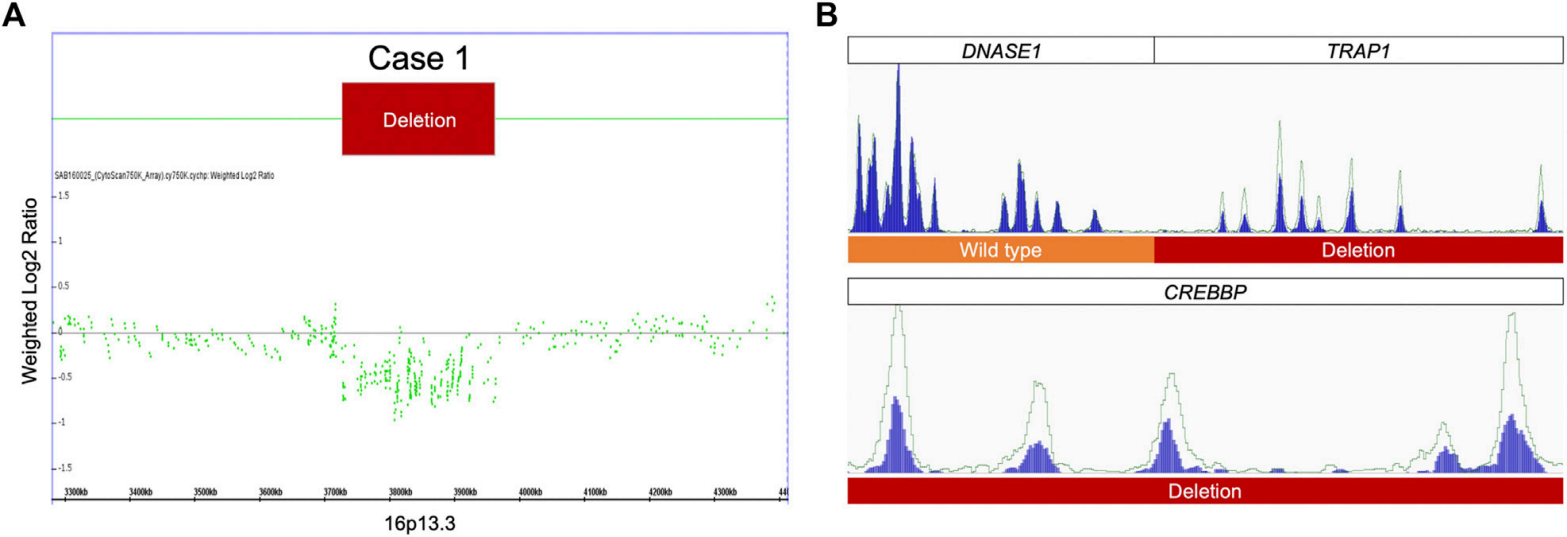
MLPA and WES results for case 1. **(A)** MLPA of the proband showing heterozygous deletion at 16p13.3. **(B)** A visualization of the WES results for case 1 show fewer reads in the proband than in the healthy control, confirming the heterozygous deletion detected by MLPA.

Case 1 was a 4-year-old female showing typical clinical features, including a high-arched palate, broadened thumbs and halluces, and a tendency to keloid development (Figure 2A). Congenital cyanotic heart disease were also found, including hypoplastic aortic isthmus with mild coarctation of the aorta, perimembranous ventricular septal defect, large right patent ductus arteriosus (PDA), and anomalous left pulmonary artery arising from left side PDA. MLPA of DNA from the proband and the parents revealed a *de novo* heterozygous 16p13.3:3,728,096-3,962,938 deletion that spanned the loci of *TRAP1* and *CREBBP* (Figures 3A, 4A). To validate the results, we also performed WES on the proband’s DNA, and a visualization of the WES results in IGV revealed low read coverage across the mutation span (Figure 4B). Surgical repair of the patient’s congenital cyanotic heart disease was performed to alleviate the cardiac symptoms, and diflucortolone ointment was prescribed for the treatment of the developed keloid. The developmental delay was managed by follow-up treatments in the outpatient department and diet education.

**FIGURE 5:**
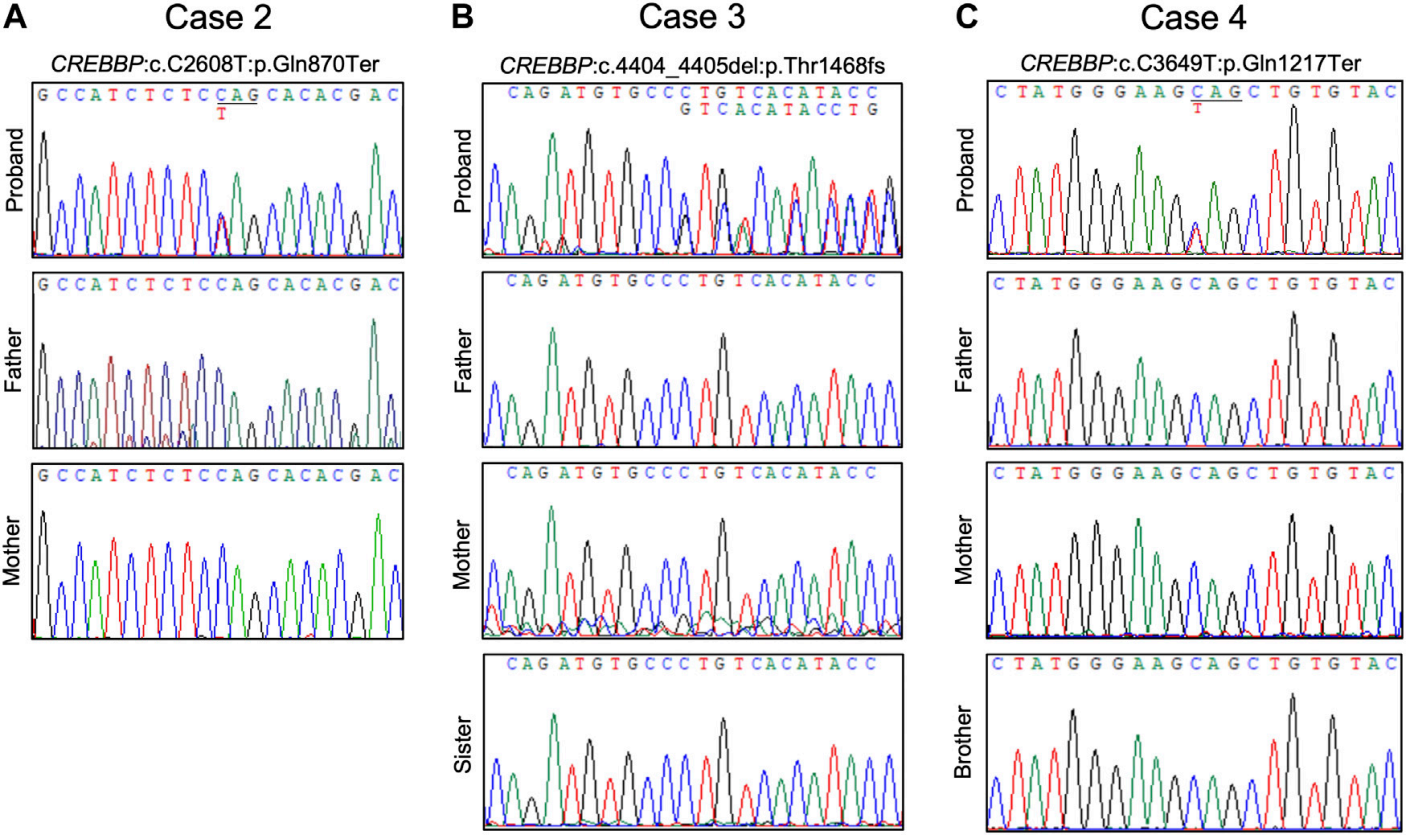
Chromatograms of confirmative PCR-based Sanger sequencing and segregation analysis results. **(A)** A *de novo* nonsense *CREBBP* mutation in case 2. **(B)** A *de novo CREBBP* 2-bp deletion in case 3. **(C)** The *CREBBP* nonsense mutation was detected in both case 4 and the proband’s mother.

Case 2 was a 15-year-old male with typical clinical features suggesting RSTS (Figure 2B). MLPA of case 2 showed no detectable deletions in *CREBBP*. WES of the proband’s germline DNA revealed a novel *CREBBP* heterozygous nonsense mutation (NM_004380:c.C2608T:p.Gln870Ter) with a significant pathogenicity score (CADD:21.4, DANN:0.9986). Further segregation analysis by Sanger sequencing confirmed the variant detected through WES and revealed both parents to harbor homozygous wild-type alleles at the mutation site, confirming that the proband’s mutation had occurred *de novo* (Figures 3B, 5A). Since that the patient also presented with intellectual disability and mental retardation, the patient was referred to neurologists and psychiatrists at our hospital for diagnostic interviews and further psychological assessments.

Case 3 was a 9-year-old female with typical clinical features suggesting RSTS (Figure 2C). MLPA did not reveal any large deletions spanning *CREBBP*. WES showed a 2-bp deletion leading to a frame-shift in the *CREBBP* locus (NM_004380:c.4404_4405del:p.Gly1469AlafsTer9), which had been reported in another RSTS patient (Murata, 2001). Segregation analysis showed a wild-type genotype for both parents at the mutation site (Figures 3C, 5B), indicating that the mutation had occurred *de novo*.

Case 4 a 10-year-old male, also showed features of RSTS (Figure 2D). No large deletions were found in the patient’s DNA by MLPA, but WES revealed a *CREBBP* nonsense mutation (NM_004380:c.C3649T:p.Gln1217Ter) with a significant pathogenicity score (CADD:44, DANN:0.9987) that had been reported in a previous RSTS patient (Saettini et al., 2020). Further segregation analysis showed that the mutation had occurred *de novo* (Figure 5C). The patient also showed microcephaly and white matter hyperintensities under magnetic resonance imaging (MRI) examination. Rehabilitation programs and psychological assessments were arranged on a regular time course for the neurological symptoms.

Case 5 was a 4-year-old boy also with typical features of RSTS (Figure 2E). MLPA showed no large deletions in *CREBBP*. Unexpectedly, WES also failed to identify pathogenic variants within the *CREBBP* and *EP300* loci (Figure 3E). Therefore, the causative genetic or genomic variant for case 5 remains unknown. Since the patient also manifested congenital glaucoma, trabeculectomy including peripheral iridectomy under the microscope was performed.

## Discussion

Of the five patients whose clinical presentation suggested RSTS, pathogenic variants in *CREBBP* were identified in four patients (80%) using an MLPA–WES genetic diagnostic workflow. However, the causative variant for 1/5 (20%) patient, remains uncertain. Among the sequencing methods used in this study, although WES efficiently detects small indels in protein-coding (exonic) DNA regions, the detection of copy number variations (CNVs), structural variants, and homologous regions of the genome remains challenging for WES. Given that pathogenic variants in RSTS comprise single-nucleotide or oligonucleotide variations as well as large deletions, genomic assays that can screen for large genomic variants (e.g., MLPA) are highly desirable. In this study, we integrated MLPA and WES in our molecular diagnostic protocol. PCR-based Sanger sequencing was also utilized to confirm the variants identified by WES. Indeed, a similar workflow has been applied to a Korean cohort (Choi et al., 2021), leading to the detection of causative variants in 80% of their patients, which is similar to our diagnostic yield.

Given that RSTS is a rare disorder arising from *de novo* mutations, the number of reported cases remain limited. In the Taiwanese population, one study reported the clinical and molecular data of 10 RSTS patients (Hou, 2005). In that study, chromosomal deletions over the 16p13 region (responsible for the *CREBBP* gene) were detected by fluorescence *in situ* hybridization (FISH). Only 30% of the RSTS patients were detected as having interstitial submicroscopic deletions in the 16p13 region. The low diagnostic rate from the use of FISH alone might lie in its ability to resolve only large DNA deletions. Thus, pathogenic single-nucleotide variants would be neglected. Similarly, among the five RSTS patients collected in our study, only 1/5 (20%) patient showed a large DNA deletion spanning *CREBBP* (case 1). Our study highlights the potential gains from harnessing various genetic assays.

Mutation hotspots in the *CREBBP* and *EP300* loci have yet to be documented. In a previous report, mutations in the highly conserved HAT domain were regarded as leading to high pathogenicity (López et al., 2018). However, no mutations resided within the HAT domain among our patients. Previous studies have further reported that frameshift mutations are the most prevalent type of mutation in RSTS patients (López et al., 2018; Choi et al., 2021). Although limited in number, more nonsense mutations (cases 2, 4) than frameshift mutations (case 3) were observed in the current study.

In our diagnostic workflow, the causative genetic alteration in 1/5 (20%) RSTS patient was not identified. Given that the pathogenesis of RSTS is not yet fully understood, the possibility remains that mutations occurring outside the *CREBBP* and *EP300* loci may contribute to the RSTS phenotype. *CREBBP* and *EP300* are both epigenetics-associated factors that alter acetyltransferase activity. In a previous study, experiments on a developmental animal model for RSTS were conducted using mice deficient in CREB-binding protein (CBP), and these CBP^+/−^ mice were found to exhibit an abnormal RSTS skeletal pattern (Tanaka et al., 1997). Furthermore, transgenic mice generated by Korzus et al. expressing reversible CBP that lacked HAT activity showed deficits in long-term memory, indicating that the HAT activity of CBP is essential for brain information processing and cognitive function (Korzus et al., 2004; Korzus, 2017). Although the genetic and epigenetic functions of *CREBBP* genes in rodents have been thoroughly investigated, the complete pathogenicity of RSTS remains to be explored.

## Conclusion

Here, we summarize the molecular genetic diagnostic progress of five patients clinically diagnosed with RSTS. Diagnostic workflow combining MLPA and WES was carried out to identify causative genetic alterations in *CREBBP* and *EP300*. Four of the five patients were found to harbor causative mutations in *CREBBP*. As the exact pathogenesis of RSTS has yet to be completely elucidated and genetic alterations in roughly 20% of the patients have yet to be found, more research is needed on the roles of genetic and epigenetic alterations in RSTS.

## Data Availability

The original contributions presented in the study are publicly available. This data can be found here: NCBI, PRJNA806385.
